# 4485 Silicone Implant Shells Increase the Rate of Proliferation of Patient-Derived BIA-ALCL Cells but Not Primary T Cells in an Engineered Biomimetic Breast Platform

**DOI:** 10.1017/cts.2020.344

**Published:** 2020-07-29

**Authors:** Ishani Premaratne, Matthew Wright, Mariam Gadjiko, Daniel Lara, Arash Samadi, Paula Ginter, Giorgio Inghirami, Kristy Brown, Jason Spector

**Affiliations:** 1 Clinical and Translational Science Center, Weill Cornell

## Abstract

OBJECTIVES/GOALS: We use a tissue engineered, biomimetic, 3D model to study the pathogenesis of breast implant-associated anaplastic large cell lymphoma (BIA-ALCL) by comparing the effect of silicone implant shell on proliferation of patient-derived BIA-ALCL to its precursor T cells within the breast microenvironment. METHODS/STUDY POPULATION: Patient-derived breast tissue was processed for component adipocytes, ductal organoids, and stromal vascular fraction. These were suspended within 50 µl of 0.3% type I collagen matrix to which was added 200,000 cells/mL of either patient-derived BIA-ALCL cells or T progenitor cells. These were then plated into 6mm wells. As a control, both BIA-ALCL cells and T progenitor cells were suspended within type I collagen alone at the same seeding density without breast components. Before plating, wells were lined circumferentially with either textured, smooth, or no implant shell. These were 1cm by 2cm pieces dissected from the whole implant. Wells were imaged using confocal microscopy over 8 days. RESULTS/ANTICIPATED RESULTS: Unstimulated T progenitor cell count showed no significant increase in any of the conditions tested. The change in cell count over 8 days was 3.85% in each condition (p = 0.3352). A Tukey’s multiple comparison test comparing each condition revealed no significant increase in cell count over 8 days for all six conditions. Notably, our previous studies have shown proliferation of BIA-ALCL cells to be significantly more robust in the biomimetic platform compared to collagen-only groups, regardless of implant shell type (p < 0.01). BIA-ALCL cells grew nearly 30% faster in textured and smooth shell biomimetic groups compared to biomimetic wells lacking implant shell. DISCUSSION/SIGNIFICANCE OF IMPACT: Towards elucidating BIA-ALCL’s etiopathology, we show that silicone implant shell has a significant effect on proliferation of BIA-ALCL cells, but not their precursor T cells. If breast implant silicone shell is not a sufficient stimulus for T cell proliferation, co-stimulatory factors are required.

